# The PodPAD project: a podiatry-led integrated pathway for people with peripheral arterial disease in the UK – a pilot study

**DOI:** 10.1186/s13047-018-0269-y

**Published:** 2018-06-04

**Authors:** Lisa Farndon, John Stephenson, Oliver Binns-Hall, Kayleigh Knight, Sally Fowler-Davis

**Affiliations:** 1 Sheffield Podiatry Services, Sheffield Teaching Hospitals NHS Foundation Trust, Sheffield, UK; 2 School of Human & Health Sciences, University of Huddersfield, Huddersfield, UK; 3 Sheffield Hallam University /Combined Community & Acute Care Group, Sheffield Teaching Hospitals NHS Foundation Trust, Sheffield, UK

**Keywords:** Peripheral arterial disease, Activity, Intermittent claudication, Quality of life, Integrated pathway, Podiatry led, National Centres for sports and exercise medicine, Public health

## Abstract

**Background:**

Peripheral arterial disease affects the lower limb and is associated with diabetes, high cholesterol, smoking and obesity. It increases the risk of cardiovascular morbidity and mortality. It can be symptomatic causing intermittent claudication, but often there are few clinical signs. Podiatrists are able to detect the presence of peripheral arterial disease as part of their lower limb assessment and are well placed to give advice on lifestyle changes to help reduce disease progression. This is important to improve health outcomes and is offered as a prevention/public health intervention.

**Method:**

We describe the clinical and patient-centred outcomes of patients attending a podiatry-led integrated care pathway in a multi-use clinic situated in a venue supported by the National Centre for Sports and Exercise Medicine in the UK. At the baseline appointment, patients were given a full assessment where symptoms of intermittent claudication using the Edinburgh Intermittent Claudication Questionnaire, foot pulses, Doppler sounds, Ankle Brachial Pressure Indices, glycated haemoglobin (HbA1c) and cholesterol levels, and smoking status were recorded. A tailored treatment plan was devised, including referral to an exercise referral service, smoking cessation programmes (if applicable) and each participant was also seen by a dietician for nutritional advice. Participants were followed up at 3 and 6 months to assess any improvement in vascular status and with each completing the EQ-5D quality of life questionnaire and a simple satisfaction questionnaire at the end of the study. As this was a complex intervention a pilot study design was adopted to evaluate if the method and outcomes were suitable and acceptable to participants the results of which will then inform the design of a larger study.

**Results:**

Data was collected on 21 individuals; 15 men (71.4%) and 6 women (28.6%) across the 6-month study period. Eleven participants were referred onto the exercise referral service; 16 participants saw the dietician for nutritional advice at baseline and had one-to-one or telephone follow-up at 3 months. Five out of 14 participants had reduced scores from baseline of intermittent claudication during the study period. No evidence for substantive changes in Doppler sounds or ABPI measurements was revealed. Quality of life scores with the EQ-5D improved in 15 participants; this was statistically significant (*p* = 0.007) with 14 participants who completed the simple satisfaction questionnaire expressing a positive view of the programme. Of the four people who were smokers, two stopped smoking cigarettes and moved to e-cigarettes as part of smoking cessation advice.

**Conclusion:**

As this was a pilot study the sample size was low, but some statistically significant improvements with some measures were observed over the 6-month study. Podiatrists are able to provide a comprehensive vascular assessment of the lower limb and accompanying tailored advice on lifestyle changes including smoking cessation and exercise. Locating clinics in National Centres for Sports and Exercise Medicine enables easy access to exercise facilities to encourage the adoption of increased activity levels, though the long term sustainability of exercise programmes still requires evaluation.

This study was reviewed and approved by London Brent Ethics Committee IRAS ID 204611 and received research governance approval from the sponsor, Sheffield Teaching Hospitals NHS Foundation Trust Research and Innovation Office STH19410.

## Background

Peripheral Arterial Disease (PAD) is a long-term condition characterised by atherosclerotic obstruction of the lower extremity arteries [1], and is a marker of patients who are at increased risk of cardiovascular events, including myocardial infarction and stroke. The incidence in the over-60s is approximately 20%; increasing with age and other factors, including: smoking, diabetes and existing coronary arterial disease [2]. Globally, 202 million people had PAD in 2010, with a 28% increase in low and moderate income countries and 13% in high-income countries in the preceding decade [3]. In the lower limb, PAD may be symptomatic with intermittent claudication or asymptomatic and can lead to, foot ulceration and critical limb ischaemia; all of which can result in amputation. Heavy smokers are four times more likely to develop intermittent claudication compared to non-smokers [4]. Intermittent claudication is also associated with reduced quality of life and depression [5].

As clinicians of the lower limb, podiatrists are able to assess patients for signs of PAD; offering treatment, surveillance, advice and follow on referral to the vascular surgery team if needed. The clinical evidence base suggests that this intervention (foot care and advice on diet, exercise and smoking) is highly effective at reducing the progression into acute care and can reduce the incidence of amputation by 60% [6]. Strict pharmacological management of cardiovascular risks specifically in people with diabetes and foot ulceration has been shown to reduce mortality rates [7]. It is recommended that all commissioners and providers should have a clear pathway for patients suspected of PAD in the UK [6].

National Institute for Health and Care Excellence (NICE) guidelines [2] recommend that people who are at risk or have symptoms of PAD, including those who have diabetes and non-healing wounds, or have unexplained leg pain should be given a full assessment. This should involve documenting the presence and severity of symptoms of intermittent claudication and rest pain, examining the feet and legs for evidence of any lesions/ulcerations, examining pulses in the lower limb by palpation and Doppler ultrasound and calculate the ankle brachial pressure index (ABPI). Diagnosis is confirmed with reduced, absent or monophasic pulse sounds with a Doppler and an ABPI < 0.9.

This study was funded to support allied health professions in public health in the UK [6] and to support podiatry assessment and treatment to prevent mortality and amputation [7]. Significant gains in wellbeing for individual patients and cost savings from preventing interventions can be achieved from improved population level health outcomes [5]. These clinical interventions align to the evidence and to the implementation of prevention strategies in podiatry services [9].

### Treatment

Early identification of both asymptomatic and symptomatic PAD allows for early intervention: slowing disease progression and decreasing the risk of lower limb amputation and cardiovascular morbidity and mortality [8]. The overall aim is to sustain or improve mobility and quality of life. Key areas to the management of symptoms include: smoking cessation, weight management and increased activity. Regular exercise can reduce cardiovascular mortality by 50% [9] and is an effective treatment for PAD [10, 11]. Other treatments include lipid modification and statin therapy, the prevention, management of diabetes (if applicable), management of high blood pressure (if applicable) and antiplatelet therapy (if required). If conservative treatments are not effective patients can develop more severe PAD where a surgical intervention in the form of angioplasty or bypass graft may be required [12]. The aim of this study was to investigate the feasibility of a podiatry-led integrated care pathway in the UK, utilising advice on diet, activity and smoking cessation for people with PAD and measuring the clinical and patient centred outcomes.

## Methods

The study was located at a UK National Centre for Sports and Exercise Medicine, in which health care clinics are situated in existing leisure/sports facilities. This allowed easy access to gym facilities for participants, if they agreed to this as part of their activity programme. Outcomes considered to be favourable were: improvement in symptoms of PAD (reduction in intermittent claudication pain if present), improved ABPI readings, increase in number of pedal pulses palpated or a change in Doppler sounds from monophasic to bi or tri-phasic, decrease in cholesterol and HbA1c levels (if applicable) and the success of any smoking cessation and activity programmes. Quality of life and patient satisfaction with the programme was also assessed**.** Patients were excluded if they were unable to give informed consent due to lack of mental capacity and if they were unable to participate in increased activity due to other co-morbidities.

Inclusion criteria: Patients who were on the podiatry service PAD caseload were purposively sampled to take part in this pilot study. Anyone wishing to take part was given an information sheet, and an appointment was arranged for a baseline assessment.

Exclusion criteria: housebound patients, patients who had poor mobility and would be unable to take part in any activity programme and patients who were not able to give informed consent. At baseline medical history was recorded, with any symptoms associated with PAD assessed using the Edinburgh Intermittent Claudication Questionnaire [13]; and absence or presence of lower limb pulses on palpation, tri-, bi- or mono-phasic lower limb pulse sounds with a Doppler and ABPIs assessed with a Dopplex Ability System®. The EQ-5D questionnaire was given to each participant to complete to assess quality of life [13]. Clinical measurements were taken by experienced research podiatrists with training in vascular assessment. Based on this assessment, the research podiatrists tailored a treatment plan, which included advice about smoking cessation and local support services to support stopping smoking and weight loss (if applicable), recommendation to the physical activity referral scheme and participants were also given the opportunity to meet with a dietician who was present in an adjoining clinic room to get advice about nutrition and weight loss (again, if applicable).

### Data collection

Several outcome measures were recorded on participants at three time points: baseline; 3 months and 6 months. Changes from baseline to 6 months were considered to be the primary comparisons of interest.

### Peripheral arterial disease

Symptoms of PAD were assessed by means of the Edinburgh Intermittent Claudication Questionnaire (EICQ). This questionnaire comprised 5 questions, all of which could be answered with a “yes” or “no”. The first question was the following screening question:

## Do you get pain or discomfort in your legs when you walk?

If participants answered “yes” to this question, they were then requested to answer the following further 4 questions:

1. Does the pain ever begin when you are standing still or sitting?
2. Do you get this pain if you walk uphill or when you hurry?
3. Do you get this pain when you walk at an ordinary pace on the level?
4. Does the pain disappear when you rest for less than 10 min?

The respective responses *Yes*, *No*, *Yes*, *Yes*, *Yes* were taken as likely indicators of the presence of intermittent claudication (IC). A score was derived for each participant with 1 point being awarded for each instance of a response corresponding to a likely indicator of the presence of IC due to PAD (excluding presence of IC due to causes other than PAD, such as spinal stenosis). Hence scores could range from 0 (no IC; given to those who answered “no” to the first question) to 5 (highly likely presence of IC) per participant.

### Pulse measurements

Left and right posterior tibial, anterior tibial, dorsalis pedis and popliteal pulse measurements were obtained from participants at each time point, amounting to 8 measurements in total per visit. A score was derived for each participant, with 1 point being awarded for each palpable pulse reading; scores could range from 0 (indicating no pulses were palpable) to 8 (all pulses were palpable).

### Doppler readings

Doppler sound readings were also taken of left and right lower limb pulses from all participants at each time point, amounting to 8 measurements in total. Sound readings were classified as triphasic, biphasic or monophasic; with triphasic considered to be the optimum status.

### HbA1c and cholesterol

Glycated haemoglobin (HbA1c) and cholesterol readings (if applicable) were recorded from the electronic patient record as near to each appointment time point as possible if these were available.

### ABPI readings

Left and right ABPI readings were obtained from each participant at each time point using the Dopplex ability. Overall values were calculated for each participant for each time point. Values < 0.9 are an indication of PAD, with lower values indicating greater severity of disease.

### Quality of life and patient satisfaction

The EQ-5D was administered to participants at each appointment. This instrument consists of 5 questions, relating to: mobility; self-care; usual activities; pain/discomfort; and anxiety/depression. All questions were 5-point Likert-style items, with a score of 1 representing no problems, and 5 representing extreme problems. Additionally, participants rated their overall quality of life on a 10-point visual analogue scale (VAS), with higher scores representing higher quality of life. A simple satisfaction questionnaire consisting of 5 questions with a free text section at the end was administered at the 6 month appointment.

### Statistical analysis

The sample was summarised descriptively; with gender, smoking status and co-morbidities reported for all patients. Additional qualitative information for participant satisfaction was also collected and summarised.

All outcome measures were summarised at each of the three measured time points. Patterns of missingness and the effect of missing data values were assessed where appropriate. Paired samples t-tests were conducted on the full EICQ scores, pulse measurements, ABPI measurements, HbA1c levels and cholesterol levels and overall quality of life scores to assess the significance of the change in these measures from baseline to the 6-month post-baseline assessment. Effect sizes and associated 95% confidence intervals were also reported for these measures.

Further analysis was conducted on the EICQ screening question to investigate any trends over time in the proportion of participants with IC. Similar procedures were conducted on the proportions of participants recording ABPI measurements in either or both legs below a critical value. Overall trends in the proportions of participants with tri-, bi- and monophasic Doppler readings were also investigated. Individual EQ-5D item scores were analysed using multivariate methods.

### Ethical permissions

Ethical and research governance approval was given prior to commencement of the project and Patient and Public Involvement (PPI) was sought from a Citizens Reference Group and a local hospital panel. Both gave feedback on the study design and patient information sheet.

## Results

Data was collected on 21 individuals; 15 men (71.4%) and 6 women (28.6%). At baseline, 4 participants (19.0%) reported themselves to be smokers; 17 participants (81.0%) were non-smokers, of these 9 were ex-smokers and 8 had never smoked.

Participants also reported a number of co-morbidities at baseline; nine (42.9%) had hypertension; 15 (71.4%) had diabetes; 4 (19.0%) had hyperlipidaemia; 4 (19.0%) had kidney disease; 3 (14.3%) had a previous stroke or transient ischaemic attack; 4 (19.0%) had ischemic heart disease (IHD) and 5 (23.8%) had a previous myocardial infarction (MI).

### Assessment of peripheral arterial disease

All 21 participants provided responses to the Edinburgh Intermittent Claudication Questionnaire (EICQ) at the baseline assessment, ranging from 0 to 5. The mean baseline score was 2.62 (SD 1.80). At the 3-month assessment, 16 participants provided responses, again ranging from 0 to 5, and the mean score was 2.38 (SD 1.59). At the 6-month assessment, 14 participants provided responses, again ranging from 0 to 5 and the mean score was 2.36 (SD 1.78).

**Fig. 1:**
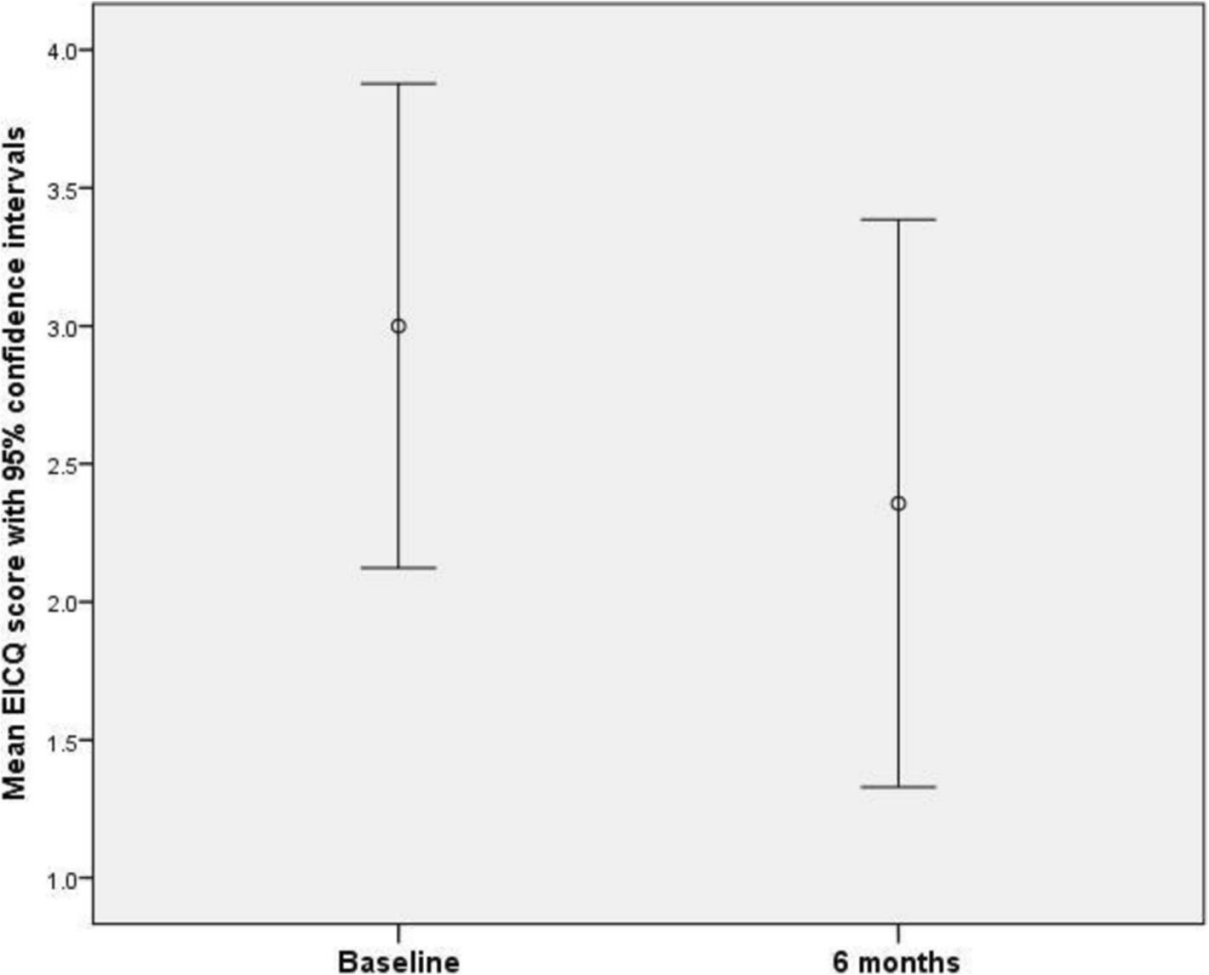
EICQ scores at baseline and at 6 months, with 95% confidence intervals

Fourteen participants provided data both at baseline and at the final 6-month assessment. The EICQ score of 5 participants reduced over this time period; whilst the EICQ score of 2 participants increased. The scores of the remaining 7 participants was unchanged. A paired samples t-test conducted on the respondents who provided data both at baseline and at 6 months found a mean reduction of 0.643 points (SD 1.34). A 95% confidence interval for the difference between baseline and 6-month data was given by (− 0.129, 1.41). The difference, though substantive, was not significant at the 5% significance level (*t*_13_ = 1.80; *p* = 0.095) (Fig. 1).

There was no evidence that missing EICQ data was not missing completely at random (MCAR) according to Little’s MCAR test (χ^2^_(3)_ = 4.41; *p* = 0.220).

Further analysis was conducted on the response to the first question alone; which was taken as a likely indicator of the presence of IC**.** At baseline, 12 participants (57.1%) were assessed to have IC. Of these 11 had other signs of PAD such as some absent pulses on palpation and or monophasic Doppler sounds. Only 1 participant had no other signs of PAD, so their pain may have been neurological in origin. Eight participants completed their 3-month assessment, of which 7 (87.5% of those completing the 3-month assessment) were assessed to still have IC. Nine participants completed their 6-month assessment (one participant who missed their 3-month assessment returned for the 6-month assessment). Of these, 6 were assessed to still have IC (66.7%) and 3 were not.

Hence overall at 6 months, 6 participants out of 12 (50.0%) were assessed to have IC; with a net change of 2 patients moving from the group with IC to the group without IC.

Of the 8 participants assessed not to have IC at baseline, 5 completed their 3-month assessment. Four were assessed to still not have IC; 1 participant was assessed to have IC at 6 months.

### Pulse measurements

All 21 participants provided pulse measurements at the baseline assessment, with scores ranging from 0 to 8 (mean 5.33 (SD 2.67)). At the 3-month assessment, 15 participants provided responses, ranging from 2 to 8 (mean 6.07 (SD 2.05)). At the 6-month assessment, 14 participants provided responses, ranging from 1 to 8 (mean 6.69 (SD 2.02)).

**Fig. 2:**
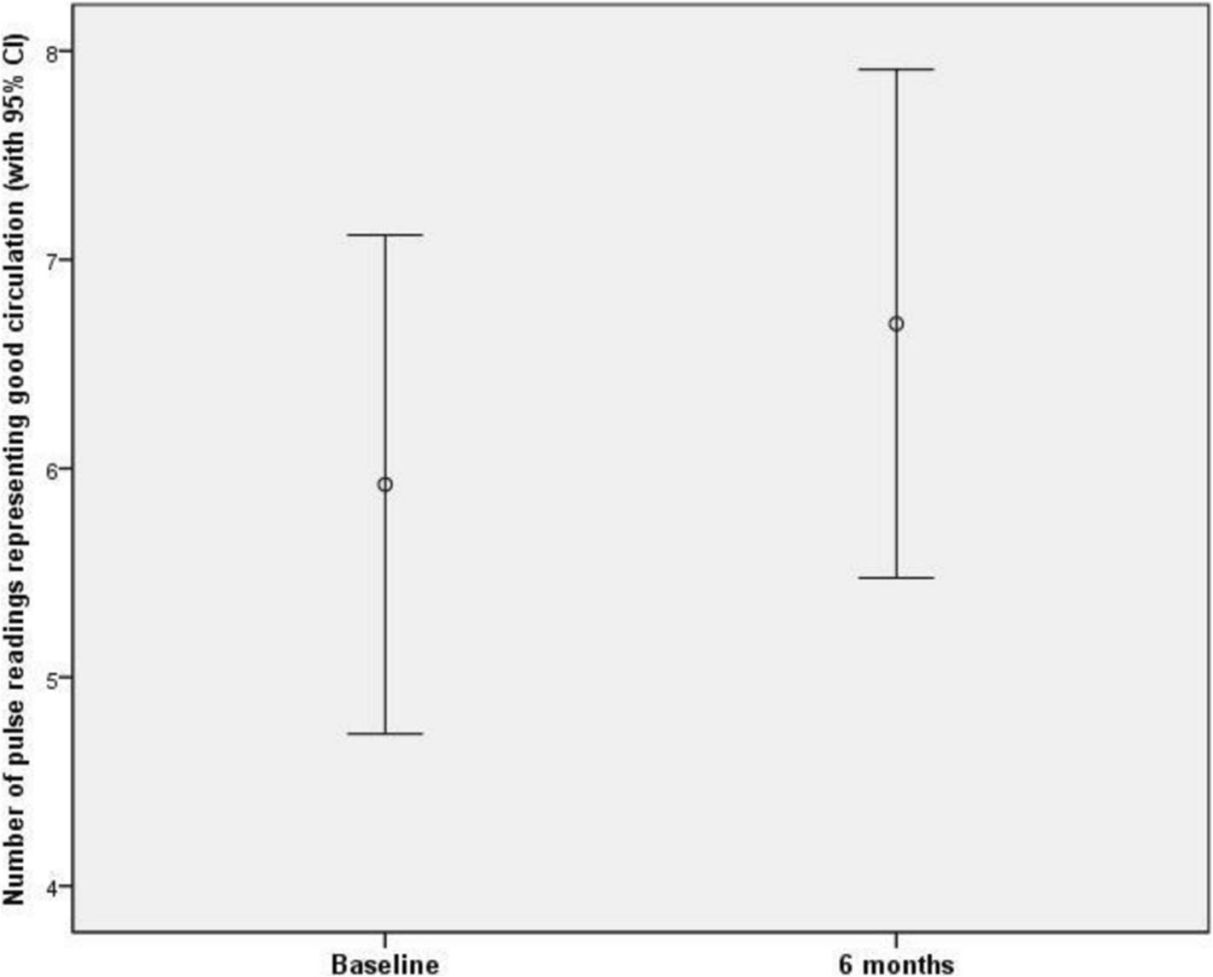
Pulse readings representing good circulation at baseline and at 6 months, with 95% confidence intervals

Thirteen participants provided pulse readings both at baseline and at the 6-month assessment. Of these, the scores of 7 participants increased over this time period; whilst scores of 2 participants reduced, with scores of 4 unchanged. A paired samples t-test conducted on the respondents who provided data both at baseline and at 6 months found a mean increase of 0.769 points (SD 2.20). A 95% confidence interval for the difference between the two time points was given by (− 0.562, 2.10). The difference was not significant at the 5% significance level (*t*_12_ = 1.26; *p* = 0.232) (Fig. 2).

### Doppler sound readings

At baseline, participants recorded on average 3.52 monophasic readings (SD 2.60); 4.38 biphasic readings (SD 2.50) and 0.10 triphasic readings (SD 0.30). At 3 months, participants recorded on average 2.47 monophasic readings (SD 2.45); 5.00 biphasic readings (SD 2.07) and 0.27 triphasic readings (SD 0.59). At 6 months, participants recorded on average 2.47 monophasic readings (SD 2.77); 5.07 biphasic readings (SD 2.69) and 0.27 triphasic readings (SD 0.59).

Hence no clear trends in recordings were observed over the three measured time points; however, the proportion of triphasic readings increased slightly from baseline to 3 months; with negligible further changes in any proportions between 3 and 6 months.

### ABPI readings

**Fig. 3:**
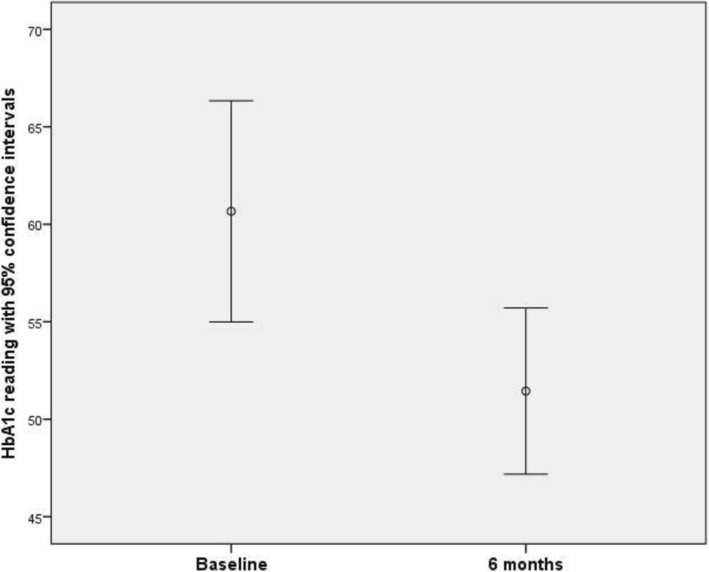
HbA1c levels at baseline and at 6 months, with 95% confidence intervals

At baseline, ABPI data was obtained from 17 participants; of whom 6 (35.3%) had overall ABPI values below 0.90 in one or both legs (range from 0.56 to 1.45). The mean value for all participants providing a reading at this time point was 0.998 (SD 0.229) in the left leg and 0.947 (SD 0.220) in the right leg. At 3 months, data was obtained from 9 participants, of whom 5 (55.6%) had overall ABPI values below 0.90 in one or both legs (range from 0.77 to 1.29). The mean value for all participants providing a reading at this time point was 0.948 (SD 0.210) in the left leg and 0.921 (SD 0.195) in the right leg. At 6 months, data was obtained from 10 participants; of which 4 (40.0%) had overall ABPI values below 0.90 (range from 0.75 to 1.20). The mean value for all participants providing a reading at this time point was 0.967 (SD 0.166) in the left leg and 0.958 (SD 0.149) in the right leg (Fig. 3).

There was no evidence that missing ABPI data was not missing completely at random (MCAR) according to Little’s MCAR test (χ^2^_(5)_ = 6.93; *p* = 0.226).

### HbA1c levels

At baseline, HbA1c data was obtained from 13 participants (range 35.0 to 75.0 mmol/l; mean 57.0 mmol/l; SD 10.6). At 3 months, data was obtained from 6 participants (range 42.0 to 70.0 mmol/l; mean 55.5 mmol/l; SD 10.7). At 6 months, data was obtained from 9 participants (range 45.0 to 63.0 mmol/l; mean 51.4 mmol/l; SD 5.55). Hence a monotonic reduction in HbA1c levels with time was apparent.

Nine participants provided data both at baseline and at the final 6-month assessment. A paired samples t-test found a mean reduction of 9.22 mmol/l (SD 7.56). A 95% confidence interval for the difference between baseline and 6-month data was given by (3.41, 15.0). The difference was significant at the 5% significance level (*t*_8_ = 3.66; *p* = 0.006). Of these 9 participants, 7 had dietetic consultation and were referred to the exercise referral service.

There was no evidence that missing HbA1c data was not missing completely at random (MCAR) according to Little’s MCAR test (χ^2^_(5)_ = 8.32; *p* = 0.140) (Fig. 3).

### Cholesterol levels

At baseline, cholesterol data was obtained from 13 participants (range 2.20 to 5.70 mmol/l; mean 3.75 mmol/l; SD 1.05). At 3 months, data was obtained from 5 participants (range 2.30 to 5.10 mmol/l; mean 3.26 mmol/l; SD 1.18). At 6 months, data was obtained from 8 participants (range 2.40 to 4.20 mmol/l; mean 3.33 mmol/l; SD 0.654). Hence no clear trend in HbA1c levels with time was apparent.

Eight participants provided data both at baseline and at the final 6-month assessment. A paired sample t-test found a mean reduction of 0.050 mmol/l (SD 0.414). A 95% confidence interval for the difference between baseline and 6-month data was given by (− 0.296, 0.369), the difference was not significant at the 5% significance level (*t*_7_ = 0.342; *p* = 0.743).

**Fig. 4:**
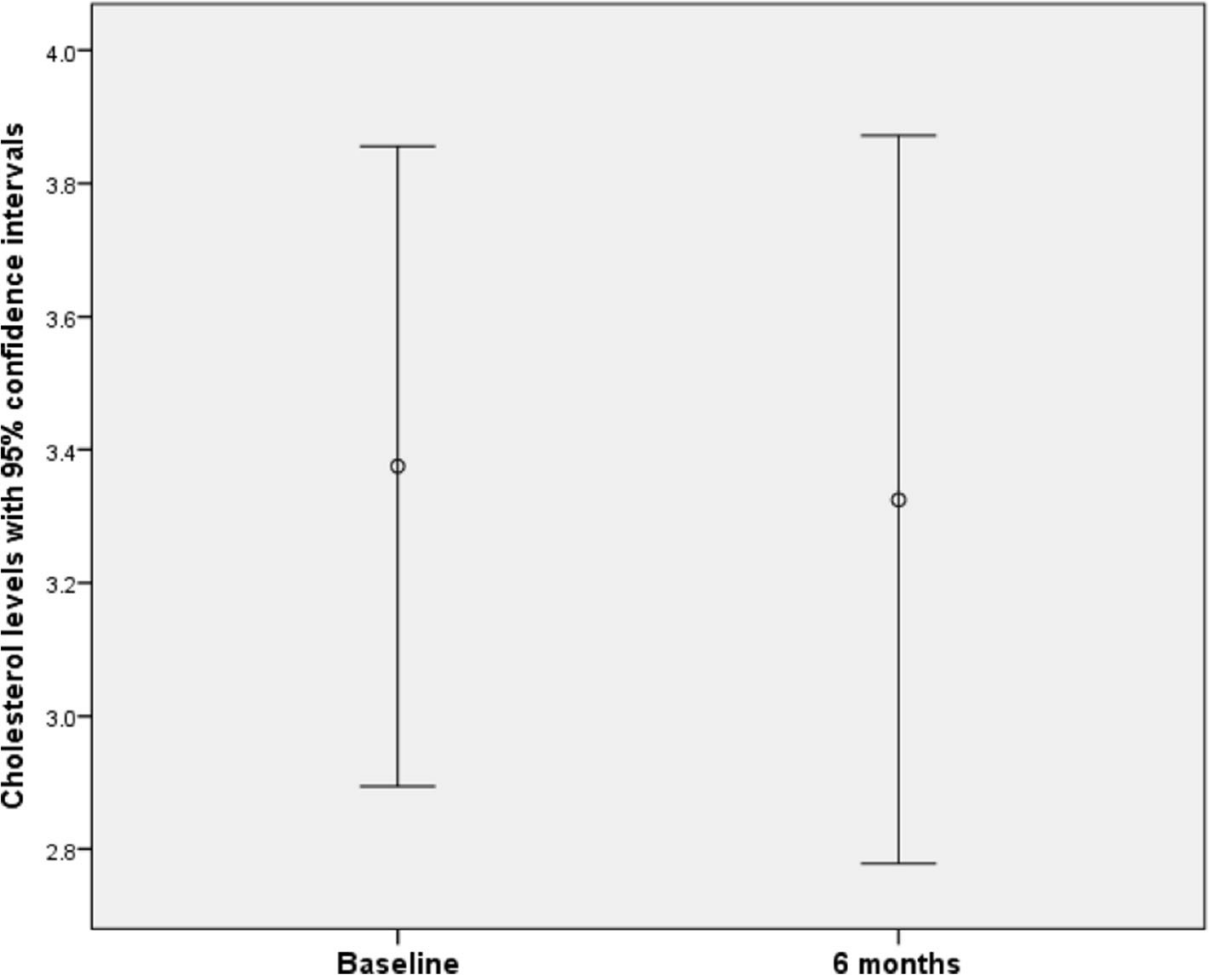
Cholesterol levels at baseline and at 6 months, with 95% confidence intervals

There was no evidence that missing cholesterol data was not missing completely at random (MCAR) according to Little’s MCAR test (χ^2^_(5)_ = 3.14; *p* = 0.678) (Fig. 4).

### Quality of life

Eighteen participants provided responses to overall EQ-5D quality of life (VAS item) at the baseline assessment (range 3.0 to 9.0; mean 5.93; SD 1.79). At the 3-month assessment, 11 participants provided responses (range 3.80 to 9.0; mean 7.34; SD 1.36). At the 6-month assessment, 15 participants provided responses (range 4.0 to 9.0; mean 7.10; SD 1.28).

**Fig. 5:**
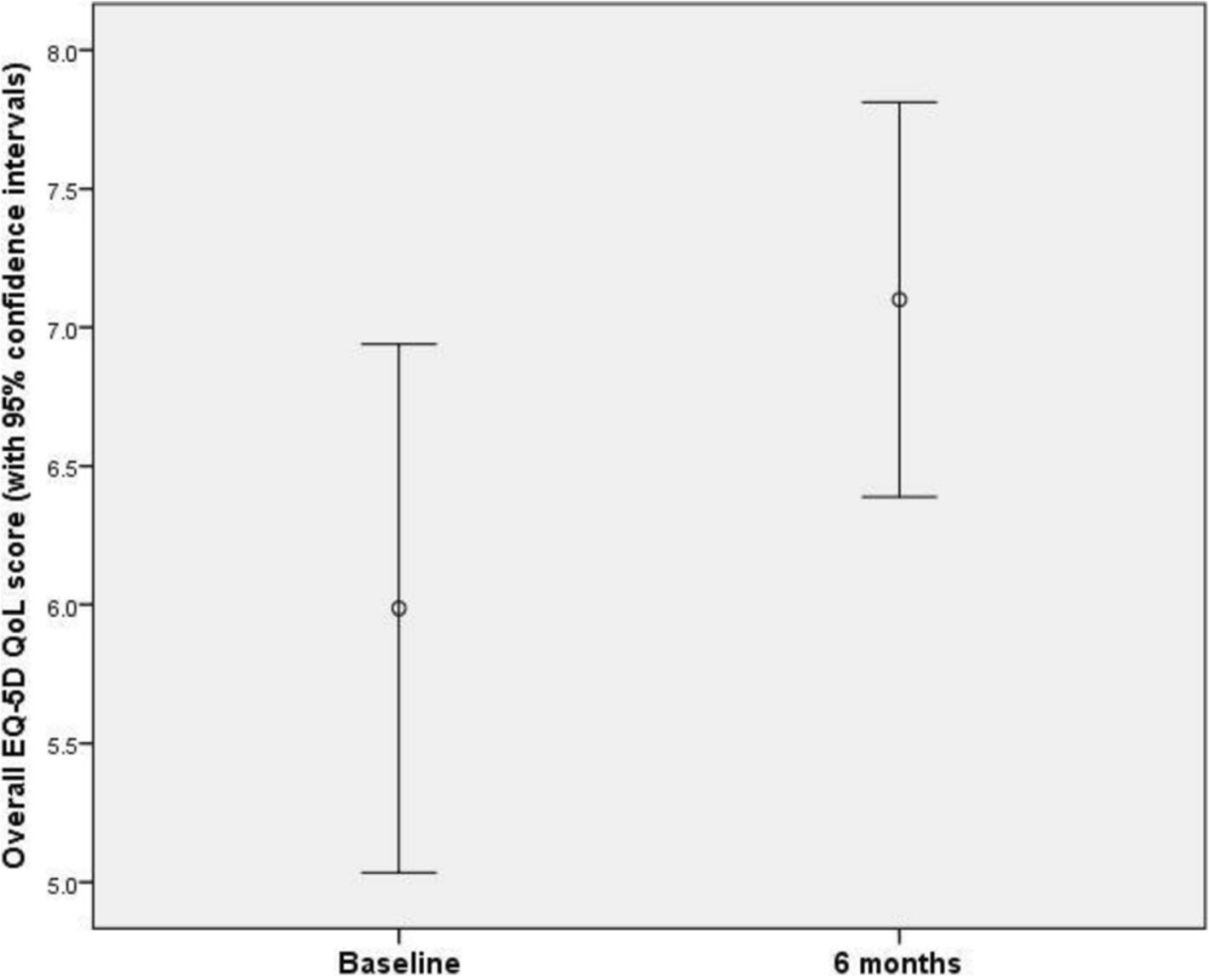
Overall EQ-5D quality of life score at baseline and at 6 months, with 95% confidence intervals

Fifteen participants provided data both at baseline and at the final 6-month assessment. A paired samples t-test found a mean increase of 1.11 points (SD 1.37). A 95% confidence interval for the difference between baseline and 6-month data was given by (0.352, 1.88). The difference was significant at the 5% significance level (*t*_14_ = 3.13; *p* = 0.007) (Fig. 5).

There was no evidence that missing overall EQ-5D data was not missing completely at random (MCAR) according to Little’s MCAR test (χ^2^_(4)_ = 6.53; *p* = 0.163). Nineteen participants provided responses to the individual item scores at the baseline, 11 at 3 months and 15 at the 6-month assessment.

**Fig. 6:**
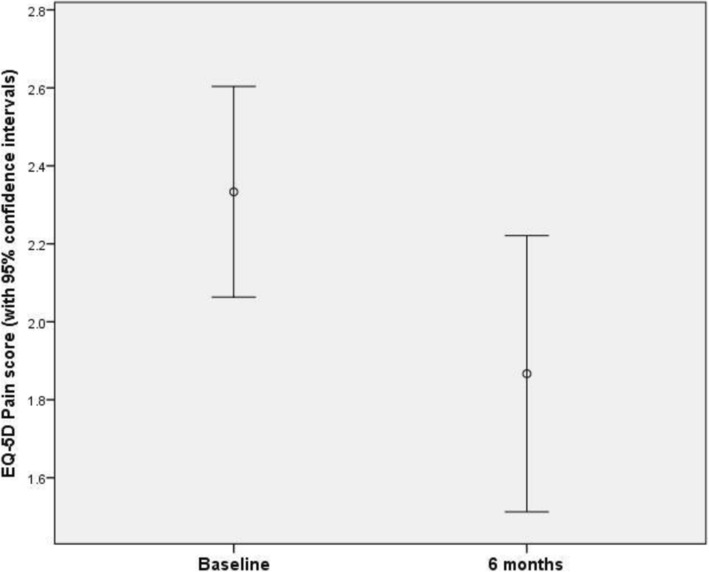
EQ-5D pain scores at baseline and at 6 months, with 95% confidence intervals

Multivariate analysis conducted on baseline data and data collected at the final 6-month assessment revealed a substantive effect (partial-η^2^ = 0.548) of time point on the measures assessed jointly (Wilk’s Λ = 0.452; *F*_4,11_ = 3.30; *p* = 0.051). Follow-up univariate analyses revealed time-related differences to be based primarily in the pain measure (*F*_1,14_ = 12.3; *p* = 0.004); with other measures not being significantly or substantively associated with the analysis time point. The reduction in pain of 0.50 points (from 2.37 points to 1.87 points) corresponded to a 21.1% reduction from the baseline value (Fig. 6).

### Other outcomes

Eleven participants agreed to be referred onto the exercise referral service situated in the same venue and 16 participants saw the dietician for nutritional advice at baseline and had one to one or telephone follow ups at 3 months. Of the four people who were smokers, two stopped smoking cigarettes and moved to e-cigarettes as part of smoking cessation advice given during the study. Seven participants had painful neuropathic symptoms at baseline and were referred either to their GP or to the painful neuropathy clinic for a medication review or further management options.

### Qualitative responses

Fourteen patients completed a simple patient satisfaction questionnaire; all of whom responded that they felt they could discuss their symptoms of PAD with the podiatrist, they felt involved with the treatment plan, they were satisfied with the service and they would recommend this service to a friend. When asked to rate their PAD symptoms after completing the programme; 9 respondents felt that they symptoms were better and the remaining 5 said that they had remained the same.

Some comments were made on individual experiences during the study, one participant said: “*I was referred to the exercise referral service, this has proved to be a dramatic change to my general wellbeing, especially the circulation in my legs.”* Another said that:

*“I have felt comfortable explaining my problems to the podiatry team and consider them to have understood and been able to diagnose where my problem lies. The information I have been given is a comfort and the advice that losing weight will help is well received. The facility and service I have found excellent with patient and friendly staff that one can be easily with in their company.”* However another participant had more difficulty: “*Struggled to complete exercises at the gym due to knee pain. Ok with arm, back and chest exercises.”*

### Limitations

As this was a pilot study there was no control group which gives some limitations on the internal validity of the study. However, the consistent and substantive improvements recorded across a wide range of outcome measures, in conjunction with lack of evidence for concurrent changes in patient lifestyles over the course of the study, provides evidence that the programme was acceptable to participants and this will inform the development of a larger study. As 7 participants had painful neuropathic symptoms at baseline, improvements in pain scores could also be associated with other interventions such as medication that was prescribed during the study period. Whilst a substantial proportion of participants were current or former smokers, the level of concurrent co-morbidities reported was not excessive; suggesting that the apparent beneficial effect of the programme has not been over-stated due to regression to the mean effects; i.e. there is no evidence that participants were skewed towards the lower end of the health spectrum to be found in the parent population, with correspondingly greater likelihood of natural improvement over time. A certain amount of attrition was observed; in general this took place between baseline and the 3-month assessment. Many patients who did not complete a 3-month assessment returned for a 6-month assessment. One patient died during the course of the study.

## Discussion

Though this was a small pilot study, some substantive, and in some cases, statistically significant improvements with respect to many measures have been observed over the 6-month study period. The extent of intermittent claudication in those participants with these symptoms (as assessed by the EICQ tool), substantively decreased, with the proportion of respondents reporting pain or discomfort when walking also decreasing. The proportion of patients providing tri-phasic Doppler sound readings increased slightly within the context of a small overall trend primarily between baseline and 3 months; with little further change beyond 3 months until the end of the study period. No substantive changes in ABPI between baseline and 6 months post baseline in either leg were revealed.

A substantive and significant reduction was recorded in HbA1c levels but no changes were observed in cholesterol levels. A significant improvement was recorded in quality of life with improvements in pain scores being mainly responsible for the overall improvement as measured by the quality of life instrument.

Previous studies have shown that patients with PAD have a much reduced level of physical activity which can be attributed to the pain associated with the disease [14]. However, if patients are persuaded to exercise or increase their activity levels, this can help with the symptoms and progression of the disease, and on-going compliance can be improved [15]. A large systematic review comparing supervised exercise with usual care for claudication found that exercise resulted in more functional benefits and should be advised as part of a treatment programme for people with PAD [16]. A Randomised Controlled Trial (RCT) comparing medical care alone, medical care plus supervised exercise and medical care plus stent revascularisation for aortoiliac PAD found that both exercise and revascularisation resulted in improved clinical outcomes and quality of life up to 18 months later [11]. Rather than a prescribed exercise programme, our study referred participants who agreed to the exercise referral service where tailored one to one advice was given to increase activity levels. This was provided on site in a National Centre for Sports and Exercise Medicine which enabled participants’ easy access to sports facilities at reduced rates.

Similarly, access to nutritional advice was made easy by the presence of a dietician on site and most participants were happy to discuss their diet as part of the programme.

Of the four patients who were smokers, two changed to e-cigarettes as part of a smoking cessation regimen. This follows current guidance from Public Health England [18] which advises e-cigarettes in conjunction with smoking cessation services. This combined approach has contributed to improved quit success rates.

The significant and substantive associations observed must be considered in the context of the study being formulated as a pilot and as such not powered to detect significant effects. Further inferences of significance may be anticipated in a larger follow-up study. Conversely, however, the large number of outcome measures has led to multiple comparisons being conducted. We have not attempted to specify a priori any outcomes as primary outcomes or correct for inflated Type I errors which would be appropriate in a full-scale study. Likewise, the size of the sample precludes the consideration of controlling factors in comparative analyses.

## Conclusion

This small, feasibility study has demonstrated important effects on health outcomes from a pilot clinical intervention for patients with PAD. The context and intervention are reported as a novel prevention-orientated treatment that sought to improve health outcomes. The findings suggest that a larger follow-up study would include a number of specific outcome measures and economic assessment to demonstrate the effect of podiatric monitoring and exercise referral for this population. Further study is needed to examine associated behaviour change factors that result from prevention services of this kind, particularly where the professional intervention is designed to mitigate the effects of chronic wounds, revascularisation and amputation, particularly with the diabetic population. The study was designed to meet the strategic vision for allied health professions to engage in specific public health and prevention interventions in the United Kingdom.

## Abbreviations

ABPI: Ankle Brachial Pressure Index
EICQ: Edinburgh Intermittent Claudication Questionnaire
HbA1c: Glycated haemoglobin
IC: Intermittent Claudication
MCAR: Missing Completely at Random
NICE: National Institute for Health and Care Excellence
PAD: Peripheral Arterial Disease
PPI: Patient and Public Involvement
SD: Standard Deviation
